# Molecular, Cytogenetic, and Immunophenotypic Biomarkers in Acute Myeloid Leukemia: Current Evidence and Future Directions—A Narrative Review

**DOI:** 10.1002/hsr2.73393

**Published:** 2026-10-13

**Authors:** Emmanuel Ifeanyi Obeagu

**Affiliations:** ^1^ Division of Haematology, Department of Biomedical and Laboratory Science Africa University Mutare Zimbabwe; ^2^ Department of Molecular Medicine and Haematology, Faculty of Health Sciences University of the Witwatersrand Johannesburg South Africa; ^3^ Department of Hematology and Immunohematology Bahir Dar University Bahir Dar Ethiopia

**Keywords:** acute myeloid leukemia, cytogenetics, immunophenotyping, molecular biomarkers, precision medicine

## Abstract

**Background:**

Acute myeloid leukemia (AML) is a highly heterogeneous hematologic malignancy with variable clinical behavior and outcomes. Conventional morphology‐based classification is insufficient to capture this complexity, necessitating the use of molecular, cytogenetic, and immunophenotypic biomarkers for accurate diagnosis, risk stratification, and therapeutic guidance.

**Objective:**

This narrative review aims to synthesize current evidence on molecular, cytogenetic, and immunophenotypic biomarkers in AML and to highlight their clinical relevance and emerging future directions.

**Methods:**

A narrative review of the literature was conducted, focusing on key molecular mutations, cytogenetic abnormalities, and immunophenotypic profiles with established or emerging diagnostic, prognostic, and predictive value in AML.

**Results:**

Molecular biomarkers such as *FLT3*, *NPM1*, *CEBPA*, *IDH1/2*, and *TP53* mutations play central roles in AML pathogenesis and prognostication. Cytogenetic abnormalities remain critical for risk classification, with favorable, intermediate, and adverse karyotypic profiles strongly influencing outcomes. Immunophenotypic biomarkers, assessed by multiparameter flow cytometry, are essential for diagnosis and minimal residual disease monitoring, providing powerful prognostic information. Integration of these biomarkers has significantly advanced precision medicine in AML.

**Conclusion:**

Molecular, cytogenetic, and immunophenotypic biomarkers collectively underpin modern AML management. Continued integration of multi‐omics data and MRD‐guided strategies is expected to further refine personalized therapy and improve clinical outcomes.

## 1 Introduction

Acute myeloid leukemia (AML) is a biologically and clinically heterogeneous hematological malignancy characterized by clonal expansion of abnormal myeloid progenitor cells, impaired differentiation, and progressive failure of normal hematopoiesis. Although AML has traditionally been diagnosed using morphology, cytochemistry, immunophenotyping, and conventional cytogenetics, advances in molecular genetics have demonstrated that morphologically similar cases may represent biologically distinct diseases with markedly different responses to therapy and clinical outcomes. Contemporary AML classification has therefore shifted from a predominantly morphology‐based system toward an integrated model in which recurrent genetic abnormalities, molecular mutations, cytogenetic features, and immunophenotypic characteristics collectively define disease identity and clinical behavior [1, 2, 3, 4]. The genomic landscape of AML is characterized by recurrent alterations involving transcription factors, epigenetic regulators, signaling pathways, tumor suppressor genes, RNA‐splicing machinery, and chromatin‐remodeling complexes. Important molecular abnormalities include mutations in NPM1, FLT3, CEBPA, TP53, IDH1, IDH2, RUNX1, ASXL1, DNMT3A, TET2, and KIT, while recurrent fusion genes such as PML::RARA, RUNX1::RUNX1T1, CBFB::MYH11, KMT2A rearrangements, DEK::NUP214, and NUP98 rearrangements define biologically and clinically meaningful AML subgroups [5, 6, 7, 8].

Cytogenetic assessment remains an essential component of AML diagnosis and risk stratification. Recurrent abnormalities such as t(8;21)/RUNX1::RUNX1T1 and inv(16) or t(16;16)/CBFB::MYH11 identify core‐binding factor AML, while complex karyotypes, monosomal karyotypes, and specific chromosome 5, 7, and 17 abnormalities are associated with adverse biological features and poor clinical outcomes [3, 4]. However, cytogenetics alone is insufficient for many patients. AML with a normal karyotype, for example, represents a heterogeneous group whose clinical behavior can differ substantially according to molecular abnormalities such as NPM1, FLT3, CEBPA, TP53, and myelodysplasia‐related gene mutations. Multiparameter flow cytometry enables confirmation of myeloid lineage, identification of aberrant antigen expression, characterization of maturation patterns, and discrimination between leukemic and normal hematopoietic populations. Antigens, including CD13, CD33, CD117, myeloperoxidase, CD34, HLA‐DR, CD38, CD56, and CD123, together with aberrant expression of lymphoid‐associated markers, can contribute to diagnostic characterization and, in selected contexts, provide information relevant to prognosis or therapeutic targeting [9, 10, 11, 12]. Therefore, this narrative review critically examines the current evidence and future directions of molecular, cytogenetic, and immunophenotypic biomarkers in AML, moving beyond a descriptive catalogue of biomarkers to evaluate their integrated clinical utility.

## 2 Narrative Methods

A narrative literature review was conducted to synthesize current evidence on molecular, cytogenetic, and immunophenotypic biomarkers in AML. Relevant literature was identified through searches of PubMed/MEDLINE, Scopus, and Web of Science, supplemented by major AML classification frameworks, clinical guidelines, pivotal clinical trials, and landmark original studies. Priority was given to recent AML‐specific evidence addressing diagnostic classification, prognostic stratification, targeted therapy, and measurable residual disease (MRD). Selected earlier studies were included where they provided foundational evidence. Evidence was critically synthesized thematically according to cytogenetic, molecular, immunophenotypic, and integrated biomarker applications, with particular attention to current limitations and emerging precision‐medicine approaches.

## 3 Mechanisms of Chemoresistance in AML

Chemoresistance in AML results from interacting genetic, epigenetic, cellular, metabolic, and microenvironmental mechanisms that permit leukemic cells to survive therapy [13, 14]. TP53 abnormalities, defective DNA‐damage responses, altered apoptotic signaling, and dysregulated BCL‐2 family proteins can reduce chemotherapy‐induced cell death [15, 16]. Increased expression of ATP‐binding cassette drug‐efflux transporters may decrease intracellular drug accumulation, while enhanced DNA repair can facilitate recovery from cytotoxic injury [17, 18]. Leukemic stem cells contribute substantially to resistance through cellular quiescence, enhanced stress responses, metabolic adaptation, and protection within specialized bone‐marrow niches. Stromal‐cell interactions, adhesion molecules, cytokines, and inflammatory signaling further promote leukemic‐cell survival [19, 20]. Metabolic reprogramming, particularly increased dependence on mitochondrial oxidative phosphorylation, may support persistence of treatment‐resistant clones. In addition, clonal heterogeneity and treatment‐driven evolution allow resistant subclones to persist or emerge during therapy [21, 22]. These mechanisms may coexist within the same patient and evolve over time, explaining why morphological remission does not necessarily represent eradication of leukemia. Integration of molecular profiling, immunophenotyping, and MRD assessment is therefore critical for identifying resistant disease and developing strategies for durable remission [23].

### 3.1 Mechanisms of Chemoresistance in AML

Chemoresistance is a major determinant of treatment failure and relapse in AML, arising from the interaction of intrinsic leukemic biology, clonal evolution, the bone‐marrow microenvironment, and treatment‐related selection pressure. At the molecular level, resistance may result from abnormalities in apoptotic signaling, DNA‐damage responses, drug transport, cellular metabolism, and survival pathways. Alterations involving TP53, for example, can impair DNA‐damage‐induced apoptosis and are associated with particularly treatment‐resistant disease, while dysregulation of BCL‐2 family proteins may allow leukemic cells to survive cytotoxic stress. Increased activity of drug‐efflux transporters, including ATP‐binding cassette proteins, can reduce intracellular concentrations of chemotherapeutic agents [13, 14, 15, 16]. Leukemic stem cells represent another important reservoir of treatment resistance because of their relative quiescence, enhanced DNA‐repair capacity, metabolic adaptations, and dependence on protective stem‐cell niches. The bone‐marrow microenvironment can further promote resistance through stromal‐cell interactions, adhesion signals, cytokine networks, and anti‐apoptotic pathways. Metabolic reprogramming, including altered mitochondrial function and oxidative phosphorylation, may support the survival of residual leukemic cells following chemotherapy [17, 18, 19]. Genetic and epigenetic heterogeneity also permits therapy‐driven clonal selection. Resistant subclones may pre‐exist at diagnosis or emerge during treatment through the acquisition of additional genomic abnormalities. Consequently, morphologic remission does not necessarily indicate eradication of resistant clones. MRD provides an important means of detecting persistent leukemia and identifying patients at increased risk of relapse. Understanding the convergence of clonal evolution, leukemic stem‐cell persistence, apoptotic escape, metabolic adaptation, drug efflux, and microenvironmental protection is therefore essential for developing strategies that prevent resistance and achieve more durable AML remission (Table 1) [20, 21, 22, 23].

**TABLE 1:** Key Molecular biomarkers in AML, their prevalence, functional impact, and clinical relevance.

Biomarker	Approximate prevalence	Functional impact	Clinical relevance
NPM1	25%–35%	Aberrant nucleophosmin localization; altered transcription and differentiation	Diagnostic classification; prognostic stratification; highly useful molecular MRD marker
FLT3‐ITD/TKD	25%–35%	Constitutive FLT3 signaling and increased proliferation/survival	Prognostic assessment; FLT3‐targeted therapy; MRD/relapse monitoring in selected settings
CEBPA	5%–10%	Disrupted myeloid differentiation and transcriptional regulation	Defines a genetically distinct AML subgroup; risk stratification
IDH1/IDH2	15%–20%	Production of 2‐hydroxyglutarate; epigenetic and differentiation abnormalities	Disease classification; targeted therapeutic options
TP53	5%–15%	Impaired DNA‐damage response and apoptosis	Adverse‐risk biology; chemoresistance; therapeutic decision‐making
RUNX1	5%–15%	Abnormal transcriptional control and myeloid differentiation	Classification and adverse‐risk assessment in appropriate contexts
ASXL1	5%–15%	Dysregulated chromatin remodeling and epigenetic regulation	Myelodysplasia‐related biology; adverse‐risk assessment
DNMT3A	15%–25%	Abnormal DNA methylation and clonal hematopoiesis	Biological classification; prognostic context; interpretation of MRD requires caution
TET2	5%–15%	Altered DNA demethylation and epigenetic regulation	Clonal evolution and disease biology; context‐dependent prognostic relevance
KMT2A rearrangement	~5%–10%	Dysregulated transcription through fusion proteins	Defines genetically distinct AML; therapeutic and prognostic implications

### 3.2 Cytogenetic Biomarkers in AML

Cytogenetic biomarkers constitute a foundational component of AML diagnosis, classification, and risk stratification. Chromosomal abnormalities reflect underlying genomic instability and define biologically distinct AML subgroups with characteristic clinical behavior and therapeutic responsiveness. Conventional karyotyping, supplemented by fluorescence in situ hybridization and molecular techniques, remains essential for identifying these prognostically significant alterations at diagnosis [19]. Balanced chromosomal rearrangements are among the most clinically informative cytogenetic biomarkers in AML. Translocations such as t(15;17), t(8;21), and inv(16) or t(16;16) are associated with favorable outcomes and distinct molecular mechanisms of leukemogenesis. These lesions disrupt key transcriptional regulators, leading to differentiation arrest while retaining sensitivity to targeted or differentiation‐based therapies. Acute promyelocytic leukemia, defined by t(15;17), represents a paradigm of cytogenetically driven precision therapy with excellent long‐term survival [20].

In contrast, patients with normal karyotype AML comprise a heterogeneous intermediate‐risk group in which molecular biomarkers are critical for further prognostic refinement. The absence of visible chromosomal abnormalities does not imply genetic simplicity but rather underscores the importance of integrating cytogenetic findings with molecular mutation profiling to accurately stratify risk and guide treatment decisions [21]. Adverse cytogenetic profiles are characterized by complex karyotypes, monosomal karyotypes, and specific chromosomal deletions or aneuploidies. These abnormalities are strongly associated with chemoresistance, early relapse, and poor overall survival. Such cytogenetic features frequently co‐occur with high‐risk molecular alterations, including *TP53* mutations, reinforcing their negative prognostic impact. Patients with adverse cytogenetics often require intensified or alternative therapeutic strategies, including consideration of allogeneic hematopoietic stem cell transplantation [22]. Cytogenetic biomarkers also have implications for disease monitoring and treatment response assessment. While less sensitive than molecular or immunophenotypic methods for minimal residual disease detection, persistent or evolving cytogenetic abnormalities during or after therapy indicate clonal persistence or evolution and portend unfavorable outcomes (Table 2) [23].

**TABLE 2:** Key cytogenetic biomarkers in AML, their prevalence, functional impact, and clinical relevance.

Cytogenetic biomarker	Approximate prevalence	Functional impact	Clinical relevance
t(8;21)/RUNX1::RUNX1T1	5%–10%	Disrupted myeloid differentiation	Defines AML entity; generally favorable‐risk biology
inv(16)/t(16;16)/CBFB::MYH11	5%–10%	Abnormal core‐binding‐factor function	Defines AML entity; generally favorable‐risk biology
t(15;17)/PML::RARA	5%–10%	Blocked promyelocyte differentiation	Diagnostic hallmark of APL; urgent differentiation therapy
KMT2A rearrangements	5%–10%	Aberrant transcriptional regulation	Defines genetic subtype; prognostic and therapeutic implications
t(6;9)/DEK::NUP214	< 2%	Abnormal transcription and nuclear transport	Distinct AML entity; generally adverse‐risk biology
inv(3)/t(3;3)/MECOM rearrangement	< 2%	Dysregulated transcription and hematopoiesis	Distinct AML entity; adverse‐risk association
BCR::ABL1	< 1%–2%	Constitutive tyrosine‐kinase signaling	Defines a rare AML subtype; requires careful diagnostic distinction
−5/del(5q)	5%–10%	Loss of tumor‐suppressive and hematopoietic genes	Adverse‐risk cytogenetic abnormality
−7/del(7q)	5%–10%	Loss of genes involved in hematopoiesis and genome stability	Adverse‐risk cytogenetic abnormality
Complex karyotype	10%–15%	Multiple chromosomal abnormalities and genomic instability	Strongly associated with adverse‐risk disease and treatment resistance

### 3.3 Immunophenotypic Biomarkers in AML

Immunophenotypic biomarkers are essential for AML lineage assignment, diagnosis, disease characterization, and MRD assessment. Multiparameter flow cytometry commonly demonstrates expression of MPO, CD13, CD33, CD117, HLA‐DR, CD34, CD38, CD64, CD14, CD11b, and CD15, with variable patterns according to AML subtype and maturation stage [23, 24]. Aberrant antigen expression, asynchronous maturation, abnormal antigen intensity, and cross‐lineage expression can distinguish leukemic cells from normal hematopoietic populations and provide patient‐specific leukemia‐associated immunophenotypes. CD34 and CD117 are frequently associated with immature blasts, whereas monocytic differentiation may be characterized by CD64, CD14, and CD11c [25, 26]. Abnormal expression of markers such as CD7 or CD19 may provide additional diagnostic and MRD targets in selected AML cases. Importantly, immunophenotypic profiles may evolve during therapy, requiring flexible MRD strategies. Integration of flow‐cytometric findings with morphology, cytogenetics, and molecular biomarkers improves diagnostic precision and longitudinal disease monitoring (Figure 1) [27, 28].

**FIGURE 1:**
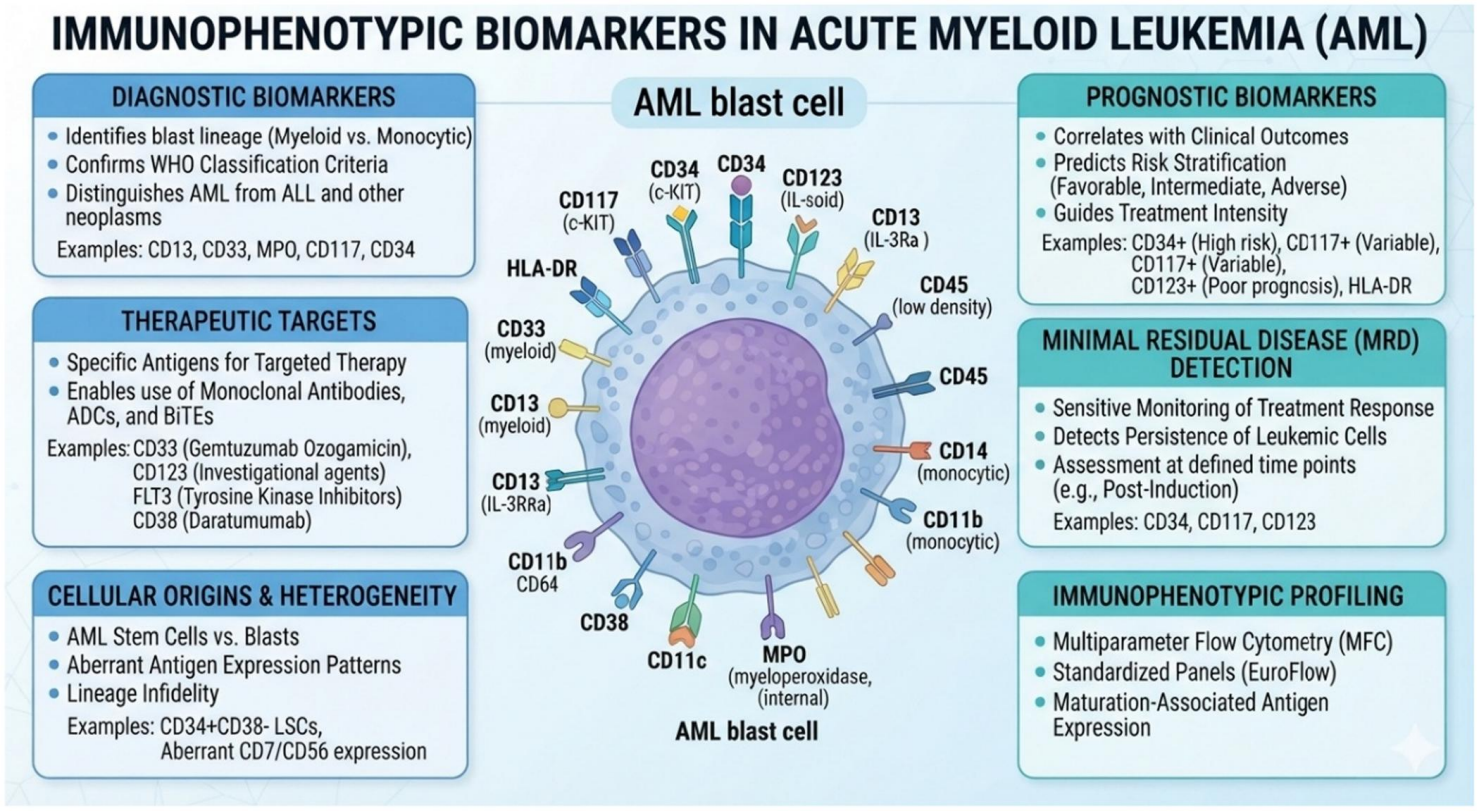
Immunophenotypic biomarkers in acute myeloid leukemia.

### 3.4 Integrated Biomarker Approaches and Clinical Implications

The integration of molecular, cytogenetic, and immunophenotypic biomarkers has fundamentally transformed the clinical management of AML. Individually, each biomarker category provides valuable but incomplete insight into disease biology. When combined, these biomarkers enable a multidimensional understanding of AML heterogeneity, allowing for refined diagnostic accuracy, precise risk stratification, and personalized therapeutic decision‐making [29]. Integrated biomarker models are now central to contemporary AML classification systems, which incorporate cytogenetic abnormalities and recurrent molecular mutations to define biologically and clinically distinct disease entities. This approach allows patients with similar morphologic features to be stratified into prognostically meaningful subgroups, thereby guiding treatment intensity and post‐remission strategies. For example, favorable‐risk patients defined by specific cytogenetic and molecular profiles may achieve durable remissions with chemotherapy alone, whereas those with adverse integrated profiles are often directed toward early consideration of allogeneic hematopoietic stem cell transplantation [30].

From a therapeutic perspective, integrated biomarker assessment informs the selection of targeted agents and combination regimens. Molecular alterations identify actionable targets, while immunophenotypic profiles confirm antigen expression relevant to antibody‐based therapies. Cytogenetic risk further contextualizes treatment response expectations and relapse risk. This convergence supports a precision medicine framework in which therapy is tailored to the biological characteristics of each patient's disease rather than a uniform treatment algorithm [31]. Integrated biomarkers are also critical for treatment response assessment and disease monitoring. MRD evaluation increasingly relies on complementary molecular and immunophenotypic techniques, offering enhanced sensitivity and prognostic power. Persistent or re‐emergent molecular or immunophenotypic abnormalities following therapy signal a high risk of relapse and may prompt preemptive therapeutic interventions. In this context, MRD‐guided treatment adaptation is emerging as a key strategy to improve long‐term outcomes (Figure 2) [32]. Despite these advances, challenges remain in standardizing integrated biomarker assessment across clinical settings. Variability in testing platforms, analytical sensitivity, and interpretation can limit comparability and clinical implementation, particularly in resource‐limited environments. Addressing these challenges will require harmonized guidelines, technological innovation, and multidisciplinary collaboration [33].

**FIGURE 2:**
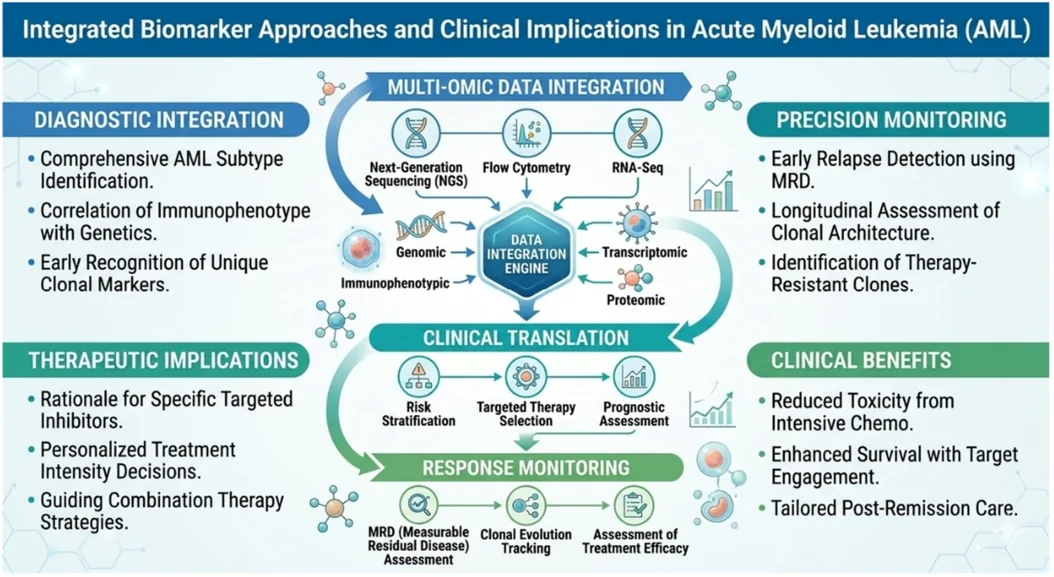
Integrated biomarker approaches and clinical implications.

### 3.5 Integrated Biomarker Signatures and Composite Prognostic Models in AML

Integrated biomarker assessment combines molecular, cytogenetic, immunophenotypic, clinical, and MRD information to provide a more comprehensive representation of AML biology and prognosis [30, 31]. Contemporary risk models increasingly incorporate recurrent genetic abnormalities such as NPM1, FLT3, CEBPA, TP53, and myelodysplasia‐related mutations alongside cytogenetic findings. Immunophenotypic characteristics complement genomic data by defining leukemic‐cell populations and supporting MRD detection [32, 33, 34]. Composite models can therefore refine risk stratification beyond individual biomarkers, particularly when multiple cooperating abnormalities are present. Importantly, prognostic interpretation is dynamic: MRD status after treatment may provide additional risk information beyond baseline genetics, while clonal evolution can alter disease biology at relapse. Future models are likely to integrate genomic profiles, flow‐cytometric and molecular MRD, clinical characteristics, and longitudinal treatment‐response data using computational and machine‐learning approaches. This multidimensional strategy supports increasingly individualized AML classification, therapeutic selection, relapse‐risk assessment, and disease monitoring (Table 3 and Table 4) [35–, 36].

**TABLE 3:** Major cytogenetic, molecular, and immunophenotypic biomarkers in AML and their clinical applications.

Biomarker category	Major biomarkers/examples	Key clinical applications
Cytogenetic	t(8;21)/RUNX1::RUNX1T1; inv(16)/CBFB::MYH11; t(15;17)/PML::RARA; KMT2A, MECOM rearrangements; −5/−7; complex karyotype	Diagnosis/classification; risk stratification; treatment planning; disease monitoring
Molecular	NPM1; FLT3; CEBPA; IDH1/2; TP53; RUNX1; ASXL1; DNMT3A; TET2	Genetic classification; prognosis; targeted therapy; MRD assessment; clonal monitoring
Immunophenotypic	MPO; CD13; CD33; CD117; CD34; HLA‐DR; CD64; CD14; CD19/CD7 aberrancy	Lineage assignment; diagnosis; leukemic phenotype characterization; flow‐MRD
Integrated biomarkers	Molecular + cytogenetic + immunophenotypic profiles	Comprehensive risk assessment; precision treatment; response and relapse monitoring
MRD biomarkers	NPM1 transcripts; fusion transcripts; leukemia‐associated immunophenotype; different‐from‐normal patterns	Treatment‐response assessment; relapse‐risk estimation; longitudinal disease surveillance

**TABLE 4:** Evolution of biomarker information across the AML disease course.

Disease stage	Key biomarker information	Clinical application
Diagnosis	Morphology, immunophenotype, cytogenetics, FISH, molecular profile	Confirm AML, establish lineage, and define genetically distinct entities
Baseline risk assessment	Cytogenetic abnormalities + NPM1, FLT3, CEBPA, TP53 and myelodysplasia‐related mutations	Assign genetic risk and estimate relapse/survival risk
Treatment selection	Actionable mutations, particularly FLT3 and IDH1/2; disease biology and patient fitness	Select targeted and risk‐adapted treatment
Early response	Morphological remission + flow‐cytometric and/or molecular MRD	Determine the depth of response and identify persistent disease
Post‐consolidation	Serial molecular MRD and multiparameter flow cytometry	Refine relapse risk and guide further treatment decisions
Post‐transplantation	Molecular/flow MRD with clinical assessment	Detect early molecular or immunophenotypic recurrence
Relapse surveillance	Serial MRD, repeat molecular profiling and cytogenetics	Detect emerging relapse and clonal evolution
Relapsed/refractory AML	New mutations, resistant clones, and altered immunophenotype	Identify resistance mechanisms and potential salvage/targeted therapies
Future precision monitoring	Integrated multi‐omics, single‐cell profiling, liquid biopsy, and AI	Dynamic prediction of relapse, resistance, and treatment response

## 4 Conclusion

Molecular, cytogenetic, and immunophenotypic biomarkers have fundamentally transformed the understanding and management of AML. Individually, each biomarker class provides critical insights into disease biology, prognosis, and therapeutic responsiveness; collectively, they enable a multidimensional, integrated framework for precision medicine. Molecular biomarkers identify recurrent mutations and actionable targets, cytogenetic alterations define biologically distinct subgroups and inform risk stratification, and immunophenotypic profiles support diagnosis, MRD monitoring, and therapeutic targeting. The integration of these biomarkers has enhanced diagnostic accuracy, refined prognostic assessment, and guided individualized treatment strategies, including the use of targeted therapies, MRD‐directed interventions, and transplantation decisions. Advances in multi‐omics technologies, high‐sensitivity MRD detection, and computational predictive modeling are expected to further personalize AML management.

## Author Contributions

**Emmanuel Ifeanyi Obeagu:** conceptualization, writing – original draft, methodology, validation, visualization, writing – review and editing, supervision.

## Ethics Statement

This study is a narrative review of previously published literature and did not involve human participants, animals, or the collection of primary data.

## Consent

The author has nothing to report.

## Conflicts of Interest

The author declares no conflicts of interest.

## Transparency Statement

The corresponding author, Emmanuel Ifeanyi Obeagu, affirms that this manuscript is an honest, accurate, and transparent account of the study being reported; that no important aspects of the study have been omitted; and that any discrepancies from the study as planned have been explained.

## Data Availability

The author has nothing to report.
